# Osteopontin Is Associated with Multiple Sclerosis Relapses

**DOI:** 10.3390/biomedicines11010178

**Published:** 2023-01-11

**Authors:** Mario Stampanoni Bassi, Fabio Buttari, Luana Gilio, Ennio Iezzi, Giovanni Galifi, Fortunata Carbone, Teresa Micillo, Ettore Dolcetti, Federica Azzolini, Antonio Bruno, Angela Borrelli, Georgia Mandolesi, Valentina Rovella, Marianna Storto, Annamaria Finardi, Roberto Furlan, Diego Centonze, Giuseppe Matarese

**Affiliations:** 1 IRCCS Neuromed, 86077 Pozzilli, Italy; 2 Neuroimmunology Unit, IRCCS Fondazione Santa Lucia, 00179 Rome, Italy; 3 Laboratory of Immunology, Institute of Experimental Endocrinology and Oncology, National Research Council, 80131 Naples, Italy; 4 Treg Cell Lab, Department of Molecular Medicine and Medical Biotechnologies, University of Naples Federico II, 80131 Naples, Italy; 5 Synaptic Immunopathology Lab, IRCCS San Raffaele, 00163 Rome, Italy; 6 Department of Human Sciences and Quality of Life Promotion, University of Roma San Raffaele, 00166 Rome, Italy; 7 Department of Systems Medicine, Tor Vergata University, 00133 Rome, Italy; 8 Clinical Neuroimmunology Unit, Institute of Experimental Neurology (INSpe), Division of Neuroscience, San Raffaele Scientific Institute, 20132 Milan, Italy

**Keywords:** osteopontin, multiple sclerosis, cytokines, inflammation, IL-6, relapses

## Abstract

Background: Osteopontin, an extracellular matrix protein involved in bone remodeling, tissue repair and inflammation, has previously been associated with increased inflammation and neurodegeneration in multiple sclerosis (MS), promoting a worse disease course. Osteopontin is also likely involved in acute MS relapses. Methods: In 47 patients with relapsing-remitting MS, we explored the correlation between the time elapsed between the last clinical relapse and lumbar puncture, and the cerebrospinal fluid (CSF) levels of osteopontin and a group of inflammatory cytokines and adipokines such as resistin, plasminogen activator inhibitor-1, osteoprotegerin, interleukin (IL)-1β, IL-2, IL-6 and IL-1 receptor antagonist (IL-1ra). We also analyzed the correlations between CSF levels of osteopontin and the other CSF molecules considered. Results: Osteopontin CSF concentrations were higher in patients with a shorter time interval between the last clinical relapse and CSF withdrawal. In addition, CSF levels of osteopontin were positively correlated with the proinflammatory cytokines IL-2 and IL-6 and negatively correlated with the anti-inflammatory molecule IL-1ra. Conclusions: Our results further suggest the role of osteopontin in acute MS relapses showing that, in proximity to relapses, osteopontin expression in CSF may be increased along with other proinflammatory mediators and correlated with decreased concentrations of anti-inflammatory molecules.

## 1. Introduction 

Inflammatory mediators play important roles in the pathogenesis and progression of multiple sclerosis (MS). Proinflammatory cytokines and chemokines are involved in MS relapses promoting the entry and activation of immune cells within the central nervous system resulting in demyelinating lesions, axonal damage, and neuronal loss. Previous studies suggest that a proinflammatory cerebrospinal fluid (CSF) milieu may be involved in disease reactivations and MS progression. Accordingly, at the time of MS diagnosis increased CSF levels of proinflammatory molecules, including interleukin (IL)-1β, IL-2, and IL-6, have been associated with higher prospective disease activity and a worse disease course [1,2,3]. 

Osteopontin is an extracellular matrix protein involved in bone remodeling, tissue repair and inflammation [4]. Osteopontin is expressed by various cell types including osteoblasts, fibroblasts, epithelial cells and immune cells such as T lymphocytes and macrophages [5,6,7]. This molecule may play an important role in the pathogenesis of MS. Higher levels of this molecule have been found in the CSF of patients with MS at the time of diagnosis [8], and increased osteopontin CSF expression has been associated with greater prospective neurodegeneration [9]. In particular, osteopontin may play a role in MS relapses and may represent a useful biomarker predicting disease activity in patients treated with DMTs [10,11]. 

To further elucidate the role of osteopontin in acute inflammatory MS activity, we explored the correlation between the relapse distance, expressed by the time interval elapsing between the last clinical relapse and the lumbar puncture (LP), and the CSF levels of osteopontin and a group of inflammatory cytokines and adipokines such as resistin, plasminogen activator inhibitor-1 (PAI-1), osteoprotegerin, IL-1β, IL-2, IL-6, IL-1 receptor antagonist (IL-1ra). 

The results showed that, among the CSF molecules analyzed, osteopontin levels correlated negatively with relapse distance. To clarify the role of this molecule in the central inflammatory milieu, we also explored the correlation between the CSF levels of osteopontin and the other cytokines analyzed.

## 2. Materials and Methods

A group of 47 patients admitted to the Neurology clinic of IRCCS Neuromed (Pozzilli, Italy) and subsequently diagnosed as affected by relapsing-remitting (RR)-MS participated in the study. Patients in whom the date of last relapse before LP could be clearly established were included. Patients with other systemic inflammatory or neurologic diseases were excluded. All patients were not treated with corticosteroids or disease-modifying therapies before CSF sampling. 

Clinical relapse was defined as the appearance of a new neurological symptom compatible with MS not associated with fever or infection, lasting at least 24 hours. Relapse distance was defined as the time interval elapsing between the last clinical relapse and LP. The clinical characteristics recorded at the time of diagnosis included: disease duration, the number of clinical relapses before LP, and clinical disability evaluated using the Expanded Disability Status Scale (EDSS) [12]. 

Radiological activity was defined as the presence of a gadolinium (Gd)-enhancing (Gd+) lesion at brain and spine MRI scan performed at the time of LP. MRI scans (1.5- or 3.0-Tesla) were performed including dual-echo proton density sequences, fluid-attenuated inversion recovery, T1-weighted spin-echo (SE), T2-weighted fast SE, and contrast-enhanced T1-weighted SE after intravenous Gd infusion (0.2 mL/kg).

CSF was collected by LP, centrifuged and then immediately stored at −80 °C. The CSF levels of osteopontin, resistin, PAI-1, osteoprotegerin, IL-1β, IL-2, IL-6, and IL-1ra were analyzed using the ProcartaPlexMix&Match Human 8-plex (Invitrogen by Thermo Fisher Scientific) in accordance with manufacturer’s instructions and expressed as picograms per milliliter (pg/mL). Fluorescence intensity was measured using Luminex^®^ 200™ system (Luminex, Austin, TX, USA), and data were analyzed with xPONENT Software Version 3.1 (Luminex). 

Kolmogorov–Smirnov test was applied to verify the normality distribution of continuous variables. Continuous data were presented as median (interquartile range, IQR = 25th–75th percentile). Categorical or dichotomous variables were presented in terms of frequency (percentage, %). Spearman’s correlation was used to assess the correlation between CSF molecules and relapse distance, and to assess possible correlations among CSF cytokines. The *p*-values were corrected for multiple testing by using the Benjamini and Hochberg method [13].

## 3. Results

The clinical characteristics of MS patients are shown in Table 1. 

We explored the correlation between relapse distance and the CSF levels of osteopontin, resistin, PAI-1, osteoprotegerin, IL-1β, IL-2, IL-6, IL-1ra.

**Figure 1:**
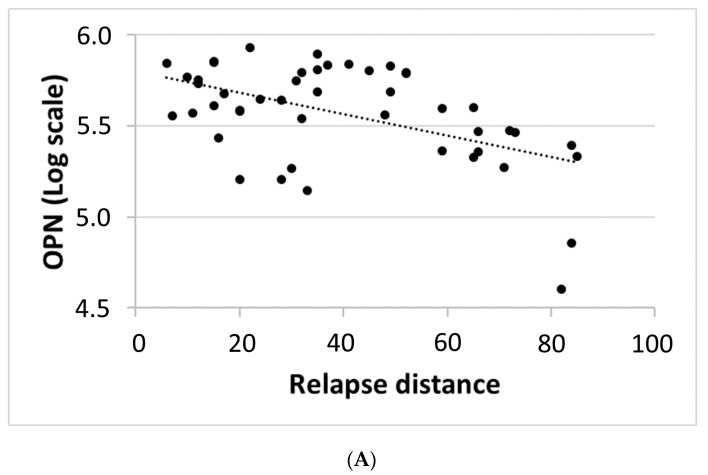

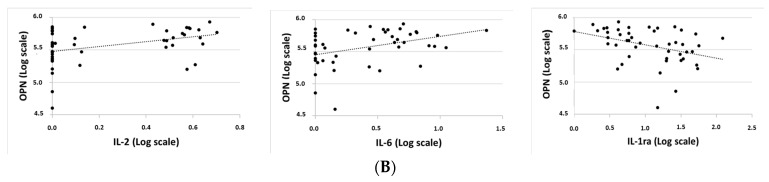
(**A**) Correlation between relapse distance and CSF molecules. Abbreviations: OPN, osteopontin. (**B)** Correlation between osteopontin and CSF cytokines. Abbreviations: CSF, cerebrospinal fluid; IL, interleukin; IL-1ra, IL-1 receptor antagonist; OPN, osteopontin.

A significant negative correlation was found between relapse distance and the CSF levels of osteopontin after correcting for multiple comparisons (Spearman’s rho= −0.392, *p* = 0.006, B-H corrected *p* = 0.048, N = 47) (Figure 1A). No significant correlations were observed between relapse distance and the other CSF molecules. In addition, no significant correlations were found between relapse distance and the clinical characteristics reported in Table 1.

No significant associations were observed between the CSF levels of osteopontin and clinical characteristics at the time of diagnosis (age, sex, OCB presence, disease duration, radiological activity, and the number of previous relapses).

To further explore the role of osteopontin in MS relapses, we analyzed the correlations between the CSF levels of osteopontin and the other CSF molecules considered. A significant positive correlation was found between osteopontin and both IL-2 (Spearman’s rho = 0.389, *p* = 0.007, N = 47, B-H corrected *p* = 0.024) and IL-6 (Spearman’s rho = 0.366, *p* = 0.012, N = 47, B-H corrected *p* = 0.028). In addition, a significant negative correlation was observed between osteopontin and IL-1ra CSF levels (Spearman’s rho= −0.447, *p* = 0.002, N = 47, B-H corrected *p* = 0.014) (Figure 1B).

## 4. Discussion

Different molecules first identified as metabolic mediators also regulate the immune system activation and have been involved in MS pathogenesis and progression [14]. Adipocytokines are a heterogeneous group of mediators with pro- and anti-inflammatory activities [15]. Some of these molecules such as osteopontin and leptin have been associated with a worse course of MS, promoting increased inflammation and neurodegeneration [8,16], and may be specifically involved in acute inflammatory MS relapses [10,17,18]. 

Here, we found a significant association between the CSF levels of osteopontin and the time interval since the last clinical relapse. Osteopontin CSF concentrations were negatively correlated with relapse distance, being higher in patients with shorter time interval between the last clinical relapse and LP. This finding may suggest a role of osteopontin in acute inflammation in MS. 

Previous studies showed that osteopontin is involved in the pathogenesis of different inflammatory and neurodegenerative diseases, including MS [19,20]. Osteopontin is released by both resident and infiltrating immune cells, promotes the activation and survival of autoreactive T lymphocytes and the production of inflammatory mediators [7,21]). Studies in animal models of MS (i.e., experimental autoimmune encephalomyelitis, EAE), evidenced that osteopontin administration induces disease reactivation [21] and osteopontin-deficient mice showed a milder disease course with decreased inflammatory infiltration, reduced expression of tumor necrosis factor and interferon gamma, and increased production of the anti-inflammatory IL-10 [22,23]. In addition, in line with a possible causal role in acute relapses, neutralizing osteopontin activity with specific antibodies promoted disease remission and improved the clinical course of EAE [24]. Higher osteopontin levels have been found in active MS lesions and in the CSF of patients with MS and other inflammatory neurological conditions [25,26]. Furthermore, increased osteopontin CSF expression has been reported in progressive MS phenotypes [8] and has been associated with greater prospective neurodegeneration in patients with MS [9]. Interestingly, increased osteopontin plasma levels have been previously reported before and during MS relapses [10]. Our results, suggesting that also osteopontin CSF expression may vary with relapse activity in RR-MS, further support the role of this molecule in acute MS relapses. 

Finally, a positive correlation was observed between the CSF levels of osteopontin and the concentrations of the proinflammatory cytokines IL-2 and IL-6. Previous studies in MS have shown an association between acute inflammatory activity and increased CSF levels of proinflammatory cytokines [3,27]. Notably, enhanced CSF expression of IL-2 and IL-6 has been reported in relapsing MS patients, [26,28] and has been associated with prospective disease activity and worse disease course [29,30]. A strong negative correlation was also observed in our study between CSF levels of osteopontin and of the anti-inflammatory molecule IL-1ra. IL-1ra, is an endogenous competitive inhibitor of IL-1β, a main proinflammatory cytokine involved in MS pathogenesis [30]. IL-1ra administration has protective effects in animal models (i.e., EAE) [31,32,33] and CSF expression of IL-1ra may affect MS course [34]. These results suggest that a proinflammatory MS milieu may be associated with acute inflammatory episodes.

Overall, our findings are in line with a role of osteopontin in acute MS relapses and suggest that, near relapses, the CSF expression of osteopontin is increased and associated with higher levels of IL-2 and IL-6 and reduced IL-1ra concentrations. Although the low number of patients and the lack of prospective data represent important limitations of the present investigation, our study reports for the first time a correlation between CSF molecules and the time elapsed since the last MS relapse. 

In the proximity of MS relapses, osteopontin expression in CSF may be increased along with other proinflammatory mediators and correlate with decreased concentrations of anti-inflammatory molecules. Modulation of osteopontin activity may represent a future target for personalized MS therapies [24]; however, factors involved in the regulation of osteopontin expression in the acute and chronic phases of the disease and during treatment with DMTs require further investigation.

## Figures and Tables

**Table 1:** Clinical characteristics of RR-MS patients.

RR-MS Patients	N	47
Age at LP, years	Median (IQR)	32.9 (23.4–41.53)
Disease duration, months	Median (IQR)	2.4 (1.17–26.13)
Sex, F	N/tot (%)	30/47 (63.8)
EDSS at LP	Median (IQR)	2 (1–3)
Radiological activity	N/tot (%)	29/47 (61.7)
OCB, yes	N/tot (%)	33/44 (75)
Number of relapses before LP *	Median (IQR) [min—max]	1 (1–2) [1–3]
Relapse distance, days	Median (IQR) [min—max]	35 (20–59) [6–85]

* including the last relapse. Abbreviations: EDSS, Expanded Disability Status Scale; LP, lumbar puncture; OCB, oligoclonal bands; RR-MS, relapsing-remitting multiple sclerosis. Missing data: OCB in 3/47 patients (6.38%).

## Data Availability

The data that support the fundings of this study are available from the corresponding author, upon reasonable request.

## Abbreviations

CSF: cerebrospinal fluid; EAE: experimental autoimmune encephalomyelitis; EDSS: Expanded Disability Status Scale; Gd: gadolinium; Gd+: gadolinium-enhancing; IL: interleukin; IL-1ra: IL-1 receptor antagonist; LP: lumbar puncture; MS, multiple sclerosis; OCB: oligoclonal bands; PAI-1: Plasminogen activator inhibitor-1; RR: relapsing-remitting; SE, spin-echo; %: frequency.
